# Awareness of personal safety among frontline healthcare workers working in COVID ward of BPKIHS during COVID-19 pandemic: a cross-sectional study authors

**DOI:** 10.1097/MS9.0000000000002319

**Published:** 2024-06-25

**Authors:** Rama Khadka, Pushpa Parajuli, Ram Sharan Mehta, Gyanand Mandal, Erina Shrestha, Pratik Adhikari, Pratik Uprety

**Affiliations:** B.P. Koirala Institute of Health Sciences, Dharan, Nepal

**Keywords:** awareness, COVID-19, frontline healthcare workers, personal safety

## Abstract

**Background:**

Frontline healthcare workers are at higher risk for COVID-19 infection and due to lack of availability of adequate personal protective equipment (PPE), lack of knowledge and good practices results in more deaths each year due to occupational accidents and diseases.

**Objective:**

The aim of the study was to assess the awareness of personal safety, the association between the level of awareness with selected socio-demographic variables and to identify the correlation between knowledge and practice of personal safety.

**Materials and methods:**

A descriptive cross-sectional study design was conducted among 106 Frontline Healthcare workers who have worked in the COVID ward. The study was conducted between 7 August 2022 and June 2023. A convenient sampling technique was used for sample selection. A validated self-administered questionnaire was used to assess the awareness of personal safety. Descriptive statistics (mean, SD frequency and percentage) and inferential statistics (χ^2^ and Spearman’s correlation rank) were used for the data analysis.

**Results:**

Among the respondents, there were 38 doctors and 68 nurses. The majority of the respondents had a moderate level of knowledge (79.2%) and practice (52.8%) with a mean score of 13.52±2.10 and 14.51± 2.35, respectively. Doctors have slightly higher levels of knowledge (14.01±1.62) and practice (14.57±2.07) as compared to Nurses (13.19±2.27, 14.48±2.5), respectively. Knowledge was found to be associated with the education level and age of the respondents, and practice has a significant association with training/demonstration with a *P* value of less than 0.05. Knowledge and practice were found to have a partial positive correlation (r value of 0.27).

**Conclusion:**

This study concluded that those having higher levels of education had good levels of knowledge and those who have attended formal or informal training or demonstrations regarding personal safety had good practices regarding personal safety.

## Introduction

Highlights

- Frontline healthcare workers face increased COVID-19 risks due to inadequate protective equipment and lack of knowledge.
- Study of 106 healthcare workers revealed moderate awareness levels in knowledge (79.2%) and practice (52.8%).
- Doctors showed slightly higher knowledge (14.01) and practice (14.57) compared to nurses (13.19, 14.48).
- Education and age linked to better knowledge; training and demonstrations associated with improved safety practices.
- Positive correlation (r=0.27) between knowledge and practice highlights the potential for enhanced safety measures.

Healthcare is one of the fastest-growing sectors of the world. A healthcare worker is one who delivers care and services to the sick and ailing either directly as doctors and nurses or indirectly as aids, helpers, laboratory technicians, or even medical waste handlers. According to the WHO, Healthcare workers are people whose job is to protect and improve the health of their communities, and whose primary intent is to enhance health. Together these health workers, in all their diversity, make up the global health workforce. According to WHO, there were ~59 million healthcare workers worldwide till 2016 among which 27.9 million were nursing workforce. In Nepal, there are 32 505 doctors (MBBS and Specialty)^1^ and 67 022 nurses (PCL and bachelor)^2^ are available to date.

Occupational safety and health (OSH) is generally defined as the science of the anticipation, recognition, evaluation and control of hazards arising in or from the workplace that could impair the health and well-being of workers, taking into account the possible impact on the surrounding communities and the general environment. This domain is necessarily vast, encompassing a large number of disciplines and numerous workplace and environmental hazards. Health workers are at risk of occupational Hazards such as biological (tuberculosis, anthrax, Human Immunodeficiency virus, hepatitis), chemical (drugs, disinfectants), ergonomic (lifting, transfers), stress/violence (staffing shortages, shift rotation), physical Hazards (radiation, heat, noise) etc^3^.

Suzy Lamplugh Trust’s definition of personal safety is “an individual’s ability to go about their everyday life free from the threat or fear of psychological, emotional or physical harm from others.”^4^

As defined by the WHO^3^ “occupational health deals with all aspects of health and safety in the workplace and has a strong focus on primary prevention of hazards.“According to the International Labor Organization (ILO), more than 2.3 million workers die every year as a result of occupational accidents or work-related diseases. All the workers have rights to occupational safety. All the work should take place in a safe and healthy working environment, and conditions of work should be consistent with workers’ well-being and human dignity, and work should offer real possibilities for personal achievement, self-fulfillment and service to society (ILO, 1984).

According to WHO, COVID-19 is an infectious disease caused by the SARS-CoV-2 viruses. It was originally identified in China, Wuhan city in 2019 and WHO declared a Public Health Emergency of International Concern on 30 January 2020 and a pandemic on 11 March 2020. COVID-19 impact has been broad, affecting general society, the global economy, culture, ecology, politics, health, education and other areas. The COVID-19 pandemic caused human loss of 5 203 303 people worldwide and 11 518 people in Nepal till 26 November 2021^5^. Among them 241 million were healthcare workers who got infected and 4.9 million have died till 20 October 2021^6^.

Every individual is at risk of developing and transmitting infection with COVID-19, especially frontline healthcare workers who are directly involved in the treatment and care of patients. In an analysis of information from the U.K. and U.S., frontline healthcare workers had a nearly 11.6 times higher risk of testing positive for COVID-19 compared with individuals in the general community^7^.

According to WHO, the COVID-19 virus can spread from an infected person’s mouth or nose in small liquid particles when they cough, sneeze, speak, sing or breathe. Most people infected with the virus will experience mild, moderate to severe respiratory illness and require medical attention. Older people and those with underlying medical conditions like cardiovascular disease, diabetes, chronic respiratory disease, or cancer are more likely to develop serious illnesses^3^. COVID-19 had a significant impact on the lives and physical and mental health of healthcare workers.

Apart from the risk of developing and transmitting COVID-19 infection, other risks that need to be considered are work time and workload, lack of recognition, including inadequate pay and support resources, prolonged use of personal protective equipment (PPE) and PPE fit, harassment, violence, stigma and discrimination, mental health, including burnout, sanitation and hygiene. Even a well-developed country like the USA faces a major challenge in managing PPE.

In an analysis of information from the U.K. and U.S., frontline healthcare workers who reported that they had inadequate access to PPE had a 23% higher risk compared to healthcare workers who had access to adequate PPE. Also, compared with healthcare workers reporting adequate PPE who did not care for patients with COVID-19, workers caring for patients with documented COVID-19 had a nearly 5-times higher risk of testing positive if they had adequate PPE and a nearly 6-times higher risk if they had inadequate PPE^7^.

WHO recommends: that contact and droplet precautions be applied during care for the patient with suspected, probable, and confirmed COVID-19. Additionally, airborne precautions are recommended to be applied during aerosol-generating procedures. WHO does not recommend: PPE reuse (donning of a used PPE item without decontamination/ reprocessing), use of gloves in settings where they are not needed, wearing a medical mask over a respirator, or the use of non-medical masks as an alternative to medical masks or respirators^3^.

In this study, our primary objective was to assess the awareness of personal safety among frontline healthcare workers working in the COVID wards of tertiary care centers during the COVID-19 pandemic.

## Methods

### Study design

**Figure 1:**
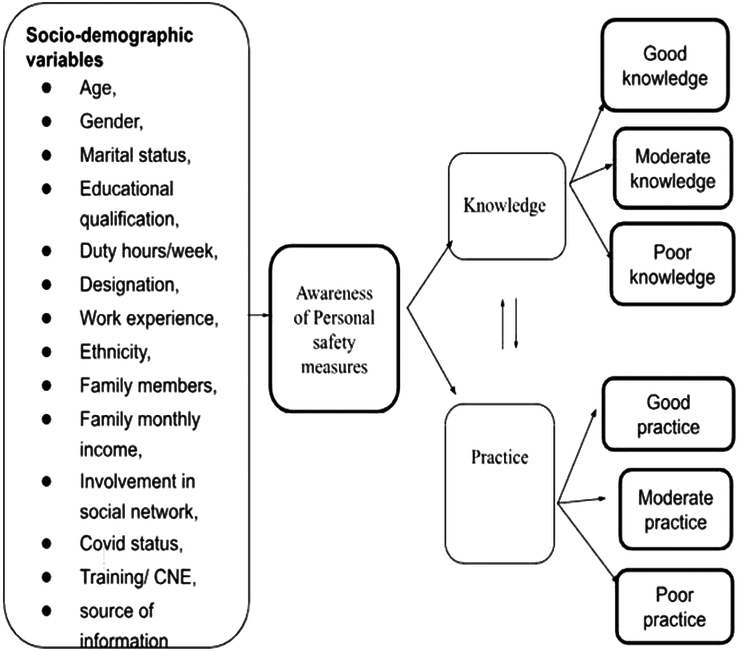
Healthcare worker personal safety questionnaire flowchart.

This study employed a descriptive retrospective cross-sectional design to analyze data on frontline healthcare workers’ practices and knowledge regarding personal safety amidst the COVID-19 pandemic. The study was conducted between 7 August 2022 and June 2023. 106 samples were taken from various wards, including the medical-surgical unit, COVID ward, pediatric unit, critical care unit, and maternity unit. A convenient sampling method recruited 106 healthcare professionals who had provided direct care to COVID-19 patients at BPKIHS for at least 1 week. This included doctors, nurses (excluding those with Auxiliary Nurse Midwife or ANM degrees), respiratory therapists, and similar roles. ANM nurses were excluded due to their potentially different training and responsibilities compared to other nurses in the COVID ward. All participants provided written consent before participating in the study. The study utilized a self-developed, self-administered questionnaire comprising demographic variables and questions of knowledge and practice related to personal safety as illustrated in Fig. 1.

### Sample size

This study has considered a 95% CI and 90% power to estimate the sample size.

According to the study “Occupational COVID-19 Prevention among Congolese Healthcare Workers: Knowledge, Practices, PPE Compliance, and Safety Imperatives.”

The mean knowledge and practice scores were 80% and 55%, respectively

Prevalence of knowledge is 80%,

Using the formula,

$$n=Z^{2}Pq/d^{2}$$

$$n={\left(1.96\right)}^{2}\times 0.80\times 0.2/{\left(0.08\right)}^{2}$$

N=96.04

Adding 10% to the calculated sample for non-response, we get

10%of96.04=9.604

N=106

Hence, 106 samples were taken for this study.

### Data collection and variables

Ethical clearance was obtained from the Institutional Review Committee, with permission from relevant authorities within the hospital setting. Written informed consent was acquired from each participant. Data collection involved the utilization of a pretested questionnaire, administered to frontline healthcare workers. The questionnaire comprised three parts: Part I encompassed socio-demographic characteristics, Part II focused on knowledge regarding personal safety, and Part III assessed practices related to personal safety. The tool underwent content validation and pretesting to ensure feasibility, completeness, comprehensiveness, and appropriateness.

### Statistical analysis

Following data collection, questionnaires were checked for completeness and coded for analysis. Data were entered into Microsoft Excel and analyzed using IBM SPSS Statistics version 11.5. Descriptive statistics, including percentages, frequencies, ranges, means, and standard deviations, were computed to describe both independent and dependent variables.

Inferential statistics were employed to explore associations between variables and identify correlations between knowledge and practice of personal safety. These tests included:

* Pearson’s χ^2^ test: Used to assess associations between categorical variables where the expected cell count is greater than 5.

** Fisher’s exact test: Used when expected cell counts were less than 5 in a χ^2^ analysis.

Statistical significance was set at *P* less than 0.05. This means that results with a *P* value less than 0.05 were considered statistically significant, indicating a rejection of the null hypothesis and a likely association between the variables.

## Results

A total of 106 respondents were included in the analysis after meeting the inclusion criteria and providing written consent. Table 1 provides an overview of these characteristics, revealing that the majority of respondents were between the ages of 26–30 years (54.7%), predominantly female (64.2%), and unmarried (62.3%). Moreover, most respondents were nurses by profession (64.2%), with a significant proportion reporting working less than or equal to 48 hours per week during COVID-19 duty (86.8%). The findings also indicate a high level of education and religious affiliation among the respondents. Table 2 shows the primary sources of COVID-19 information for respondents. The majority obtained information from news media (20.6%) and social media platforms (19.7%), with only 15.2% relying on official international health organization sites like WHO and the Centers for Disease Control and Prevention (CDC).

**Table 1:** Socio-demographic characteristics of the respondents (*n*=106).

Characteristic	Category	No. Respondents	Percentage
Age in years	21–25	23	21.7
	26–30	**58**	**54.7**
	>30	25	23.58
Median =28			
Sex	Male	38	35.8
	Female	**68**	**64.2**
Marital status	Married	40	37.7
	Unmarried	**66**	**62.3**
Religion	Hindu	**96**	**90.6**
	Others (Buddhist, Christian, Kirat)	10	9.4
Profession	Doctor	38	35.8
	Nurse	**68**	**64.2**
Duty h/week	≤48 h/week	**92**	**86.8**
	>48 h/week	14	13.2
Median =46 h/week			
Total exposure in Covid ward (weeks)	≤20 weeks	**87**	**82.1**
	>20 weeks	19	17.9
Median =5.5 weeks			
Experience	<5years	**50**	**47.2**
	5–10 years	**50**	**47.2**
	>10 years	6	5.6
Median =5 years			
Living with family	Yes	40	37.7
	No	**66**	**62.3**
If yes, presence of risk group [elderly (≥60 years) or pregnant or children (≤5 years) or members with co-morbid conditions] in family	Yes	19	47.5
	No	**21**	**52.5**
Time spends on social media	<3 h	38	35.8
	3–5 h	**48**	**45.3**
	>5 h	20	18.9
Median =3.5 h			
Covid status during Covid duty	Positive	**69**	**65.1**
	Negative	37	34.9
Training/ Demonstration	Yes	**75**	**70.8**
	No	31	29.2
View regarding preparedness of the institution to manage COVID outbreak sufficiently	Yes	**57**	**53.8**
	No	48	45.3

Bold values refer to the higher number of respondents.

**Table 2:** Source of information of the respondents (*n*=106).

Description	Frequency	Percentage
Official international health organization sites and media e.g. WHO, CDC	64	15.2
Official government sites and media e.g. Ministry of Health and population, Nepal	63	14.9
News Media e.g. TVs, radios, Magazines, Newspapers	87	20.6
Social Media e.g. WhatsApp, Facebook, Twitter, Instagram	83	19.7
Friends/family/acquaintances	66	15.6
Academic trainings/continue in service education	44	10.4
Course taught at university	15	3.6

CDC, Centers for Disease Control and Prevention.

The study assessed the knowledge scores of respondents regarding personal safety, as presented in Table 3. Notably, all respondents correctly identified the causative organism of COVID-19, while a substantial percentage demonstrated awareness of COVID variants and diagnostic testing protocols. However, gaps in knowledge were observed regarding specific precautions, such as the need for additional airborne precautions during certain medical procedures. Additionally, Table 4 highlights respondents’ understanding of transmission modes, clinical symptoms, and complications of COVID-19, with varying levels of accuracy across different domains.

**Table 3:** Knowledge of the respondents regarding personal safety (*n*=106).

Knowledge items	Correct response, *n* (%)	Incorrect response, *n* (%)
Causative organism of COVID-19	106 (100)	0
Variant of corona virus which causes severity	65 (61.32)	41 (38.67)
Most affected age group by alpha variant of COVID-19	36 (33.96)	70 (66.03)
Incubation period of COVID-19	47 (44.33)	59 (55.66)
Diagnostic tests to diagnose COVID−19 infections	91 (85.84)	15 (14.15)
Spread of disease in sub-clinical stage	87 (82.07)	19 (17.92)
Use of all components of PPE while providing care to COVID-19 patients	104 (98.11)	2 (1.88)
Use of additional airborne precaution while drawing venous sample of COVID infected patient	14 (13.2)	92 (86.8)
Use of additional airborne precaution while performing aerosol generating procedures	97 (91.5)	9 (8.4)
Hand hygiene along with PPE for protection from COVID-19	101 (95.2)	5 (4.71)
Filtration value of medical masks and respirator masks (N95, N99, FFP2 etc.)	24 (22.64)	82 (77.35)
Knowledge about fit test	27 (25.47)	79 (74.52)
Percentage of alcohol in hand-sanitizer	33 (31.1)	73 (68.86)
Hand wash time with soap and water	44 (41.50)	62 (58.49)
Duration for which corona virus remain in the air	17 (16.03)	89 (83.96)
Time of highest risk of virus dispersion	85 (80.1)	21 (19.81)

PPE, personal protective equipment.

**Table 4:** Knowledge of the respondents regarding personal safety (*n*=106).

Knowledge items	Components of the items	Frequency	Percentage
Source of infection of COVID-19	An infected person	102	67.5
	Contaminated water/utensils/instruments	49	32.5
Modes of transmission of COVID-19	Droplet transmission	98	29.3
	Close contact with an infected person	92	27.5
	Touching contaminated surfaces	76	22.7
	Airborne transmission	69	20.6
Clinical symptoms of COVID-19	Sore throat	92	13.3
	Cough	88	12.7
	Runny nose	77	11.1
	Fever	96	13.9
	Loss of taste and smell	96	13.9
	Shortness of breath	84	12.2
	Body-ache and headache	91	13.2
	GI symptoms like diarrhea and vomiting	67	9.7
Complications of COVID-19	Acute Respiratory Distress Syndrome (ARDS)	99	52.4
	Septic shock	45	23.8
	Multiple organ Failure	45	23.8
Disposal of a used mask	After it becomes moist	37	28.7
	After a single use	92	71.3
Moments of hand-washing	Before touching patient or doing any procedure	97	36.6
	When hand feels dirty	8	3
	After touching contaminated surface/ objects/ patient	86	32.5
	After touching or shaking hands with others	74	27.9

GI, gastrointestinal.

Practices related to personal safety among respondents were also evaluated, as shown in Table 5. The majority of respondents reported adhering to standard donning and doffing procedures for PPE and WHO-recommended hand hygiene practices. However, certain areas of improvement were identified, including the correct disposal of used masks and maintaining social distancing both inside and outside the hospital premises. Furthermore, Table 6 illustrates respondents’ practices in preventing COVID-19 transmission. Key practices include frequent hand washing and sanitizer use (21.2%), providing masks to suspected patients (17.7%), routine disinfection of surfaces (18.4%), and using respirator masks (62.9%). Additionally, 13.6% prioritized adequately ventilated rooms for COVID-19 patients.

**Table 5:** Practice of the respondents regarding personal safety (*n*=106).

Practice items	Correct response, *n* (%)	Incorrect response, *n* (%)
Follow standard donning and doffing of PPE practices	99 (93.4)	7 (6.6)
Remove jewelry/mobile or other personal belongings before donning of PPE	98 (92.5)	8 (7.5)
Practice hand hygiene/disinfect hands following WHO 5 moments of hand-washing	101 (95.3)	5 (4.7)
Perform a fit test before using N95 or equivalent masks	41 (38.7)	65 (61.3)
Use of same mask	69 (65.09)	37 (34.9)
Using reusable PPE during COVID duty	85 (80.2)	21 (19.8)
Avoid social activities while experiencing flu like symptoms	77 (72.6)	29 (27.4)
Practicing social distancing of at least 1 m (3.28 feet) inside and outside duty area	41 (38.7)	65 (61.3)
Maintain quarantine with family and other people	93 (87.7)	13 (12.3)
Use of goggles and/or face-shield during COVID patient care	96 (90.6)	10 (9.4)
Use of shoe cover during COVID patient care	92 (86.8)	14 (13.2)
Use of a designated area/room for doffing of PPE	100 (94.3)	6 (5.7)
Use bio-hazard bags during doffing	83 (78.3)	23 (21.7)
Bath before going home from COVID duty	106 (100)	0
Sanitize accessories immediately before leaving for home	98 (92.5)	8 (7.5)
Arrange WHO 7 steps of handwashing in sequential orders.	53 (50)	53 (50)
Arrange the steps of donning of PPE in sequential order	28 (26.4)	78 (73.6)
Arrange the steps of doffing of PPE in sequential order	32 (30.2)	74 (69.8)

PPE, personal protective equipment.

**Table 6:** Practice of respondents regarding personal safety (*n*=106).

Practice items	Components of the items	Frequency	Percentage
Practice to prevent transmission of COVID-19	Frequent hand washing and use of alcohol-based sanitizers	103	21.2
	Putting masks on suspected COVID-19 patients	86	17.7
	Protective clothing and masks to healthcare workers	86	17.7
	Routine disinfection of surfaces that comes in contact of COVID-19 cases	89	18.4
	Placing COVID-19 patients in adequately ventilated rooms	66	13.6
	Avoid unnecessary moving of patients	55	11.3
Type of mask used	Surgical mask	49	37.1
	Respirator mask (N95, FFP2, FFP3 or equivalent)	83	62.9

The study further explored the association between knowledge and practice of personal safety with selected socio-demographic variables, as depicted in Tables 7 and 8. Significant associations were found between knowledge and education level, as well as the age of the respondents. Moreover, the practice of personal safety was significantly associated with training or demonstration received during COVID-19 duty.

**Table 7:** Association between knowledge of personal safety and selected socio-demographic variables (*n*=106).

		Knowledge, *n* (%)		
Characteristics	Categories	Poor	Moderate/good	*P*
Age in years	≤25	5 (21.7)	18 (78.3)	**0.04** *
	>25	6 (7.2)	76 (92.8)	
Sex	Male	0	38 (100)	0.07 **
	Female	11 (16.2)	57 (83.8)	
Marital status	Married	6 (15)	34 (85)	0.224 **
	Unmarried	5 (7.6)	61 (92.4)	
Educational level	Diploma (PCL)	10 (19.6)	41 (80.4)	**0.003** **
	Graduated	1 (1.8)	54 (98.2)	
Ethnicity	Janajati	7 (18.9)	30 (81.1)	0.47 **
	Others (Brahmin, Chettri, Dalit, Newar, Madhesi)	4 (5.8)	65 (94.2)	
Religion	Hindu	10 (10.4)	86 (89.6)	1 **
	Others (Buddhist, Christian, Kirat)	1 (10)	9 (90)	
Profession	Doctor	0	38 (100)	0.07 **
	Nurse	11 (16.2)	57 (83.8)	
Duty h/week	≤48 h/week	10 (10.9)	82 (89.1)	1**
	>48 h/week	1 (7.1)	13 (92.9)	
Total exposure in Covid ward (weeks)	≤20 weeks	11 (12.6)	76 (87.4)	0.207 **
	>20 weeks	0	19 (100)	
Experience	<5	5 (10)	45 (90)	0.904*
	≥5	6 (10.7)	50 (89.3)	
Living with family	Yes	5 (12.5)	35 (87.5)	0.577*
	No	6 (9.1)	60 (90.9)	
Time spends on social media	<3 h	3 (7.9)	35 (92.1)	0.743
	≥3 h	8 (11.8)	60 (88.2)	
Covid status				
During Covid duty	positive	9 (13)	60 (87)	0.322**
	Negative	2 (5.4%	35 (94.6)	
Training/Demonstration	Yes	6 (8)	69 (92)	0.292*
	No	5 (16.1)	26 (83.9)	

Bold values refer to the higher number of respondents.

* Pearson χ^2^ test, significant *P* value ≤ 0.05.

** Fisher’s exact test.

**Table 8:** Association between practice of personal safety and selected socio-demographic variables (*n*=106).

		Knowledge, *n* (%)		
Characteristics	Categories	Poor	Moderate/good	*P*
Age in years	≤25	0	23 (100)	0.58**
	>25	5 (6)	78 (94)	
Sex	Male	0	38 (100)	0.15**
	Female	5 (7.4)	63 (92.6)	
Marital status	Married	3 (7.5)	37 (92.5)	0.36**
	Unmarried	2 (3)	64 (97)	
Educational level	Diploma (PCL)	3 (5.9)	48 (94.1)	0.67**
	Graduated	2 (3.6)	53 (96.4)	
Ethnicity	Janajati	3 (8.1)	34 (91.9)	0.34**
	Others (Brahmin, Chettri, Dalit, Newar, Madhesi)	2 (2.9)	67 (97.1)	
Religion	Hindu	4 (4.2)	92 (95.8)	0.39**
	Others (Buddhist, Christian, Kirat)	1 (10)	9 (90)	
Profession	Doctor	0	38 (100)	0.15**
	Nurse	5 (7.4)	63 (92.6)	
Duty h/week	≤48 h/week	5 (5.4)	87 (94.6)	1**
	>48 h/week	0	14 (100)	
Total exposure in Covid ward (weeks)	≤20 weeks	5 (5.7)	82 (94.3)	0.5#
	>20 weeks	0	19 (100)	
Experience	<5	1 (2)	49 (98)	0.36**
	≥5	4 (7.1)	52 (92.9)	
Living with family	Yes	2 (5)	38 (95)	1**
	No	3 (4.5)	63 (95.5)	
Time spends on social media	<3 h	2 (5.3)	36 (94.7)	1**
	≥3 h	3 (4.4)	65 (95.6)	
Covid status during Covid duty	positive	4 (5.8)	65 (94.2)	0.65**
	Negative	1 (2.7)	36 (97.3)	
Training/Demonstration	Yes	1 (1.3)	74 (98.7)	**0.02** **
	No	4 (12.9)	27 (87.1)	

Bold values are statistically significant.

* Pearson test.

** Fisher’s exact test.

# Significant p value ≤ 0.05.

Table 9 illustrates a partial positive correlation between knowledge and practice of personal safety, indicating that an increase or decrease in one variable tends to correspond with a similar change in the other. This finding underscores the importance of enhancing both knowledge and practice to improve overall personal safety awareness among frontline healthcare workers.

**Table 9:** Correlation between knowledge and practice of personal safety.

	Mean±S.D	r value	*P*
Knowledge	13.52±2.10	**0.27**	0.004
Practice	14.51±2.35		

Bold value shows correlation between knowledge and practice.

**Figure 2:**
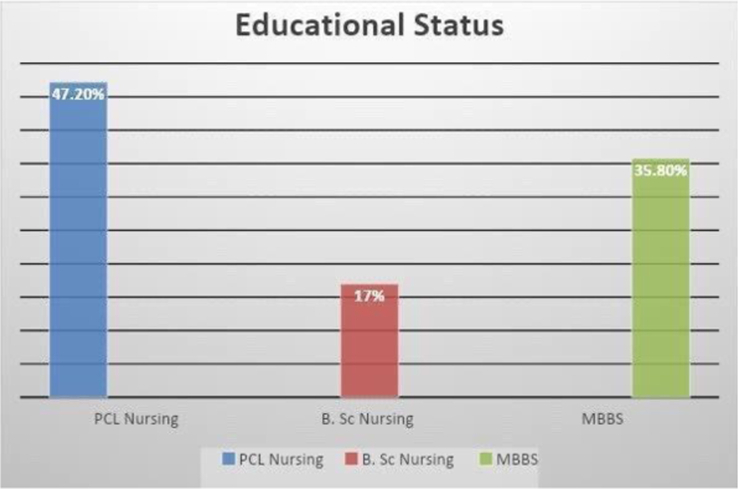
Educational status of the respondents (*n*=106).

**Figure 3:**
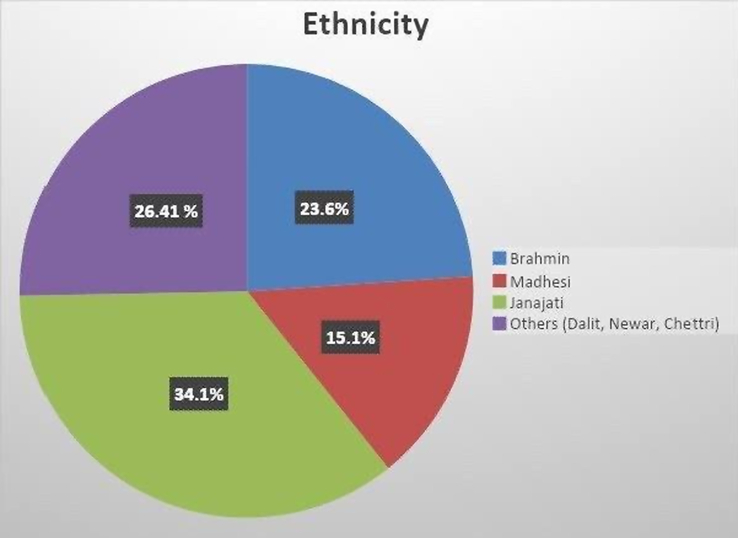
Ethnicity of the respondents (*n*=106).

**Figure 4:**
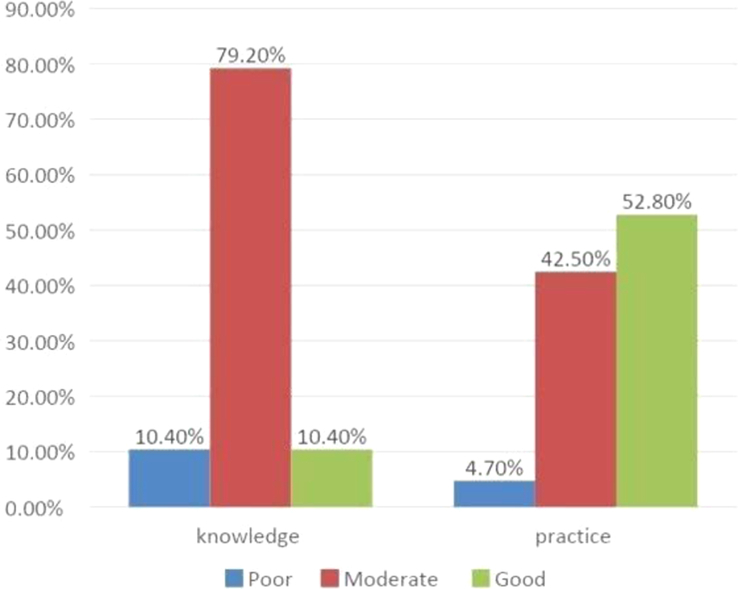
Cut-off for awareness (knowledge and practice) of the respondents regarding personal safety (*n*=106).

Lastly, Fig. 2 depicts the educational status of the respondents (*n*=106), providing insights into their academic background. Figure 3 illustrates the ethnicity distribution among the respondents, shedding light on the diverse cultural representation within the sample. Additionally, Fig. 4 presents the cut-off for awareness concerning knowledge and practice of personal safety among the respondents (*n*=106), offering a threshold for evaluating their understanding and implementation of safety measures.

## Discussion

In this study, we investigated the awareness of personal safety among frontline healthcare workers in COVID wards of tertiary care centers during the pandemic and explored associations with selected demographic variables and the correlation between knowledge and practice.

Our study included 106 participants (38 doctors and 68 nurses) selected through convenient sampling. The majority of respondents were 26–30 years old (54.7%)^8^, female (64.2%)^9^, unmarried (62.3%), and had educational backgrounds in nursing (47.2% PCL nursing, 35.8% MBBS, and 35.8% B.Sc Nursing)^10^.

Regarding work hours and experience, most respondents worked less than 48 hours per week (86.8%) and had less than 20 weeks of exposure in the COVID ward (82.1%)^11^. Interestingly, 65.1% of respondents tested positive for COVID-19 during duty^15^.

Regarding training, 70.8% of respondents received formal/informal training on personal safety during COVID duty, primarily through hospital initiatives and online resources^12^.

In terms of information sources, social media (45.3%) and news media (20.6%) were commonly used, with fewer relying on official international health organization sites (15.2%) or government sources (14.9%)^13^.

Assessment of awareness showed that 79.20% of respondents had moderate knowledge levels, and 52.80% demonstrated good practice levels^12^. The correlation analysis revealed a positive relationship between knowledge and practice (R=0.27, *P*=0.004)^15^, indicating that higher knowledge levels corresponded to better safety practices or vice versa.

Notably, significant associations were found between knowledge levels and education (*P*=0.003) and age (*P*=0.04) of respondents^12^. Similarly, practice levels were associated with training/demonstration received (*P*=0.02)^12^, reinforcing the importance of targeted training programs.

While these findings align with previous studies in some aspects, such as training associations with practice, variations exist due to contextual differences among countries and healthcare systems. Understanding these nuances can guide tailored interventions to enhance safety practices among frontline healthcare workers during pandemics.

### Strengths and limitations of the study

The study benefits from several strengths, including its comprehensive data collection process utilizing a validated questionnaire, which facilitated a thorough assessment of awareness levels among frontline healthcare workers. With a sample size of 106 healthcare workers, comprising both doctors and nurses with experience in COVID wards, the study captured diverse perspectives, enhancing the generalizability of findings to similar settings. Moreover, the use of appropriate statistical analyses, such as descriptive statistics and correlation tests, allowed for meaningful insights into the factors influencing awareness levels and practices among participants.

However, the study is subject to several limitations that warrant consideration. Firstly, the use of convenience sampling may introduce selection bias, potentially limiting the representativeness of the sample and the generalizability of findings to broader healthcare contexts. Additionally, reliance on self-reported data through questionnaires could introduce response bias, leading to inaccuracies or overestimations of knowledge and practices regarding personal safety. Lastly, the cross-sectional design of the study provides only a snapshot of awareness levels and practices at a specific point in time, hindering the assessment of the long-term effectiveness of interventions and causal relationships between variables. Despite these limitations, the study’s strengths contribute valuable insights into the awareness and practices of frontline healthcare workers during the COVID-19 pandemic.

## Conclusion

Based on the findings, the study concluded that more than a fourth of the respondents have a moderate level of knowledge and more than half of them had a good level of practice regarding personal safety. Doctors have a slightly higher level of knowledge and practice in comparison to nurses.

Similarly, knowledge was found to have a significant association with the education and age of respondents, and practice was found to be associated significantly with training/demonstration. The knowledge and practice of personal safety were found to be partially positively correlated.

The findings of the study can provide a basis for investigations for other investigators.

I am writing following the STROCCS 2021 guidelines^20^.

## Ethical approval

Ethical clearance for this study was obtained from the Institutional Review Committee (IRC) with reference number 185/078/079.

## Consent

Participants in the study were provided with detailed information about the research and voluntarily consented to participate. Written informed consent was obtained from each participant, ensuring comprehension and acknowledgement of their right to withdraw. The study protocol and research tools were approved by the Institutional Review Committee (IRC), and participants’ confidentiality and rights were upheld throughout the study.

## Source of funding

None.

## Author contribution

R.K.: writing: original draft of manuscript, resources, review and editing, conceptualization, editing, data curation, visualization, investigator. P.P.: validation, project administration, review and editing, supervision. R.S.M.: validation, review and editing, supervision. G.N.M.: review and editing, supervision. E.S.: review and editing, supervision. P.A.: writing: original draft of manuscript, resource, data curation, visualization, investigator. P.U.: resources, data curation, investigator.

## Conflicts of interest disclosure

The author declared no relevant financial conflict or any other conflict of interest.

## Research registration unique identifying number (UIN)

This is a case report involving a human subject, so registration of research study was done.

1. Registry used: Researchregistry.com.
2. Unique Identifying number or registration ID: researchregistry9426.

## Guarantor

Mr. Pratik Adhikari is the guarantor of the research study.

## Data availability statement

The datasets supporting the conclusions of this article are included within the article.

## Provenance and peer review

Not commissioned or externally peer-reviewed.
